# 10 Steps to Strategically Build and Implement your Enterprise Imaging System: HIMSS-SIIM Collaborative White Paper

**DOI:** 10.1007/s10278-019-00236-w

**Published:** 2019-06-08

**Authors:** Henri Primo, Matthew Bishop, Louis Lannum, Dawn Cram, Abe Nader, Roger Boodoo

**Affiliations:** 1 Primo Medical Imaging Informatics, Inc, Chicago, IL 60646 USA; 2 UnityPoint Health, Des Moines, IA USA; 3 AGFA Healthcare, Mortsel, Belgium; 4 The Gordian Knot Group, LLC, Fort Lauderdale, FL USA; 5 Medical Imaging & Medical Imaging Informatics, Inova Health System, Falls Church, VA USA; 6 Diagnosis Protocol & Clinical Informatics Fellow, University of Illinois Hospital and Health Sciences System, Chicago, IL 60612 USA

**Keywords:** Data Capture, Data Integrity, Electronic Health Record, Enterprise Digital Image Archive, Enterprise Image Viewing, Enterprise Imaging, Governance, Image Data Management, Imaging Informatics, PACS, Universal Viewer, Workflow

## Abstract

An enterprise imaging (EI) strategy is an organized plan to optimize the electronic health record (EHR) so that healthcare providers have intuitive and immediate access to all patient clinical images and their associated documentation, regardless of source. We describe ten steps recommended to achieve the goal of implementing EI for an institution. The first step is to define and access all images used for medical decision-making. Next, demonstrate how EI is a powerful strategy for enhancing patient and caregiver experience, improving population health, and reducing cost. Then, it is recommended that one must understand the specialties and their clinical workflow challenges as related to imaging. Step four is to create a strategy to improve quality of care and patient safety with EI. Step five demonstrates how EI can reduce costs. Then, show how EI can help enhance the patient experience. Step seven suggests how EI can enhance the work life of caregivers and step eight describes how to develop EI governance. Step nine describes the plan to implement an EI project, and finally, step 10, to understand cybersecurity from a patient safety perspective and to protect images from accidental and malicious intrusion.

## Goal

This white paper (WP) is designed to help care providers and informatics professionals champion enterprise imaging (EI) at their local healthcare enterprise. The content that follows will help these enterprise imaging advocates to build a presentation to convince C-suite executives (such as the Chief Executive Officer, Chief Financial Officer, Chief Medical Officer, Chief Informatics Officer, and Chief Medical Informatics Officer) and departmental directors or other leadership of the importance of enterprise imaging. The WP can even be used to build a strategic plan for the design and implementation of an enterprise imaging program. Some sections in the document have a technical orientation, especially when addressing mechanisms and instruments in EI. This technical content is not important for the C-level but it helps EI Advocates to have a deeper insight into these matters.

## Overview

Enterprise imaging (EI) has been defined as “a set of strategies, initiatives and workflows implemented across a healthcare enterprise to consistently and optimally capture, index, manage, store, distribute, view, exchange, and analyze all clinical imaging and multimedia content to enhance the electronic health record.” [1]

An EI strategy is an organized plan devised to optimize the electronic health record (EHR) so that healthcare providers have intuitive and immediate access to all patient clinical images and their associated documentation, regardless of source. Healthcare providers must be able to both contribute image and video content to the electronic health record and access images and video created by other clinic appointments and procedures, such as radiology, cardiology, pathology, dermatology, ophthalmology, point of care ultrasound, and endoscopy. Leveraging EI capabilities enables a number of important imaging-based use cases that will be explained in this paper. How precise are written textual descriptions of skin lesions in the field of dermatology, retinal damage in ophthalmology, and wound conditions written by the wound care team? Words can only paint a subjective mental picture. Supplementing descriptive text with images better conveys a precise depiction of the area of interest. [2]

The following 10 steps address different benefits and requirements of an EI strategy.

## Step 1: Access to all the Images and Documentation for Better Decision-Making to Impact Patient Outcomes

In contrast with radiology and cardiology, where images are created by X-ray apparatus, magnetic resonance (MR) imaging, computed tomography (CT), or ultrasound (US) scanners, most images produced outside these departments are actually images in the visible light spectrum (VL), not in the X-ray, MR, or US wavelength spectrum. VL imaging may serve evidentiary and documentation purposes. Departments such as, but not limited to, dentistry, pathology, dermatology, wound care, GI endoscopy, surgery, and others use VL in their care workflow in addition to X-ray, MR, or US. Today, these images are often not available through the EHR and are seldom securely digitally archived. This can be a challenge to patient care and patient experience, the caregiver’s quality of work, costs containment, and value-based care delivery. An effective EI strategy can help.

Examples

- Images can be used in a store-and-forward workflow to consult with other specialists; the archived images can be compared over different time points for comparisons. Images from radiology and cardiology are usually represented by the Digital Imaging and Communications in Medicine (DICOM) format. Even if these VL images are created in a non-DICOM format such as jpeg or PDF, e.g., with point-and-shoot cameras, smartphones, flatbed document scanners, medical cameras, microscopes, or pathology slide scanners, these images can be encapsulated in a DICOM object after image capture. In other words, the images can be compliant with the DICOM standard and stored in the Enterprise Vendor Neutral Archive (VNA). Also, many VNA brands can consume non-DICOM images in their native format.
- The ability to view any image from a single viewer via the EHR system with a single login at the point of care.
- Availability of historical images during the interpretation of a current image can improve the quality of interpretation.
- Diminish inappropriate utilization (duplicate exams) by making prior and new images easily accessible.
- Diminish radiation exposure to the individual patient and general population by avoiding unnecessary exam duplication.
- Not having to navigate multiple departmental systems to locate the imaging information will motivate the caregiver to view not only the patient’s clinical information in the EHR but to also view images from many sources.

### Ubiquitous Image Access

Integrated Delivery Networks (IDN) have resources and service lines working in multiple sites and locations, such as different hospitals, imaging centers, and ambulatory care offices. The information technology infrastructure needs to support this distributed organization model by enabling enterprise-wide consolidation of tools and a paperless workflow. Work should be automatically allocated to resources who are appropriately credentialed to do the work and who have sufficient bandwidth to handle the work. This can only be granted by a system that enables access independent from where and how the data was gathered.

Consolidation of systems is a best-in-class process when IDN’s acquire more institutions and expand the EHR system into these new locations. With an EI strategy in place, caregivers across the IDN can now have access to the patient’s EHR and imaging information combined. Enabling caregivers with accurate and precise information, anytime and anywhere, is a powerful tool to enable improvements in patient care. Further, a decrease in cost can be expected by avoiding unnecessary duplication of resources such as local image storage management systems in different departments in different geographical locations. Collaboration between primary care and specialty physicians in different IDN locations can be facilitated. This effect is already experienced by the early adopters of EI.

## Step 2. Demonstrate how EI Is a Powerful Strategy

The Triple Aim—enhancing the patient experience, improving population health, and reducing costs—is widely accepted as a compass to optimize health system performance [3]. The industry is moving towards a Quadruple Aim, adding the goal of improving the work life of healthcare providers, including clinicians and staff due to workload burnout. EI has the potential to help organizations achieve Quadruple aim. This includes:

1. Enhancing patient and caregiver experience: As hospitals and health systems move to entirely EHR-based (centric) models, a patient-centric view of all clinical information (including images), from within the EHR and at the point of care, enables the care provider to make quick, informed, and accurate clinical decisions. An EI strategy of combined image and EHR access eliminates the care provider’s frustration with searching for images in several departmental IT systems. EI enables quicker diagnosis and treatment allowing the provider to spend more time with the patient. It also allows the provider to keep the patient informed with accurate information, including images, about their condition and treatments. This can give the patient a sense of confidence that they are indeed receiving the best care available.
2. Improving population health: Imaging analytics and artificial intelligence applications can provide the information needed (population surveillance) to facilitate preventative programs to manage the health of populations. Mammography, colonoscopy for colon cancer, abdominal aortal aneurysm, and low-dose CT lung screening are good examples. The importance of having these data available in the EHR is well recognized. Adding the evidence images to the textual information increases the value of the information in the EHR for Population Health Management (PHM) staff. Further, the EI VNA provides a centralized and straightforward data access point for both clinical and business analytics tools.
3. Reducing costs: By consolidating imaging data into a single (or fewer) repositories substantial financial savings can be realized through consolidation of existing hardware and storage systems. Centralized data management will reduce maintenance costs and reduce or eliminate the need for future data migrations.

## Step 3: Understand the Specialties and Their Clinical Workflow Challenges as It Relates to Imaging

There are at least four types of imaging data created and consumed in the healthcare enterprise as part of clinical workflows.

1. Traditional imaging data: The first (and most common) is the imaging data created in radiology and cardiology. This data is typically created by X-ray, CT, ultrasound, nuclear medicine, and MR devices. The data created here are well defined and normalized to correspond with the data in the EMR. The systems used to manage the imaging data are enterprise class picture archiving and communication systems (PACS) and provide good integration with the EMR for “in context” viewing of the images by providers from multiple specialties.

- Radiology is an example of a specialty that creates images and reports data primarily for use outside of the specialty. Examples of specialties that consume radiology studies for diagnosis and pre-treatment or pre-surgical planning include orthopedics, oncology, obstetrics and gynecology, emergency medicine, and surgery.
- Cardiology is an example of a specialty that creates images and reports primarily use within the specialty. Examples of sub-specialties within cardiology that use imaging data are diagnostic cardiology, interventional cardiology, electrophysiology, cardiac ultrasound (echocardiography), and cardiac surgery.

2. Visible light imaging data: The second (and rapidly growing) imaging data type is the visible light imaging. This data is created by a wide variety of specialties and devices as already mentioned earlier. The data created here are typically managed by small, department level systems. The images are not usually available for viewing outside the system they were created on and are not well integrated to the EMR.

- Endoscopy is an example of a tool to create visible light imaging data as part of the procedure. These images are often combined with other imaging modalities for hybrid imaging data sets. These data sets consist of visible light images combined with either ultrasound or X-ray images. Examples would include endobronchial ultrasound bronchoscopy or endoscopic retrograde cholangiopancreatography procedures. Reports are often communicated to patients or other providers in a printed paper format with text and images creating an additional imaging document that will need to be stored and managed to be available from within the EHR.
- Dermatology, wound care, and plastic surgery, general surgery, and ophthalmology primarily create visible light images through traditional or specialty photography or video. These specialties are a good example of imaging data sets that cross specialties to complete a course of treatment for a patient.

Example: A patient may have a wound that is caused by a dermatological condition. Treatment of the condition could involve collaboration between a dermatologist and wound care physician. Once the wound is sufficiently healed, a plastic surgeon may be engaged to perform a reconstructive procedure. The images captured during this episode of care would be useful to all three-specialty areas and should be easily accessible to all three to facilitate quality care with a minimum amount of diagnostic noise created by dissimilar imaging systems.

3. Multimedia imaging data: The third source of imaging data is multimedia documents. These are typically created by department level systems such as endoscopy management and pulmonary function test systems. They can also be created by combining textual result data from the EMR with imaging data from multiple sources.

- Multimedia documents are stored in various locations depending on the system that created them. These multimedia documents could be stored in a number of external locations (PACS, document management system, etc.) based on the systems configuration or requirements or remain in the system that created them. This makes it difficult for caregivers to locate and view the various documents in a format that enables clinical comparison.

4. Waveforms: The fourth source of imaging data is waveforms. These include electrocardiography and electroencephalogram graphs, pressure-volume curves, and flow diagrams. Written reports with waveforms are often stored in departmental systems. Making these reports available with the waveforms through the EHR makes these documents easier to consume

The EI advocate should visit all the departments which use imaging in their daily practice and observe how these departments have embedded imaging in their respective workflows. Then, the EI advocate should analyze the workflow, discuss with the department the issues with the current workflow and look for possible improvements to the acquisition, storage, retrieval, and display of data.

## Step 4: Create a High-Reliability Healthcare Strategy to Improve Quality of Care and Patient Safety with EI

Having all patient images and associated reporting data easily accessible from within the EHR and at the point of care is essential for the quality of care. Imaging data combined with the textual and discrete data in the EHR can present a complete longitudinal view of the patient’s situation.

1. For patients and providers, centralized access to patient data is critical for making quick, well informed, and accurate clinical decisions that improve both patient safety and the quality of the care they receive. Combining images with textual data provides a comprehensive view of patient information.
2. For health information management (HIM), having centralized access to all patient information, including the images can make release of information (ROI) much easier while requiring less effort to gather the information.
3. For population health management, providing a consolidated, single, and easily accessible view of the entire patient record allows access to a view of patient information that has already been normalized to a single standard. This makes the job of the analytics team easier by providing accurate data with fewer opportunities to corrupt or lose data through efforts to normalize across multiple sources of data.

## Step 5: Demonstrate how EI Can Reduce Costs

Having a robust EI (EI) infrastructure in place enables the creation of new or revised workflows in multiple clinical and operational areas. The nature of the ROI’s to be harvested will most likely be different for different healthcare delivery organizations. Some examples include the following:

1. ROI could come from improved workflows in the different disciplines and the associated gains in efficiency and effectiveness.

- ROI could be the result of a consolidated IT infrastructure by eliminating local departmental IT infrastructure. Maintenance and technical services could be consolidated.
- An EI infrastructure, through its centralized storage and management of imaging data, can reduce duplicate imaging studies performed on a patient by enabling sharing of imaging data both internally and externally.
- The ability to ingest and normalize imaging studies from other facilities, with different systems and data formats, into local systems and formats can reduce the need for additional (duplicate) imaging studies.

2. Traditional workflows like radiology and cardiology can be modified and improved using the EI infrastructure. This could include global worklists based on specialty, geography, criticality, or any number of clinical or operational factors enabling the optimum use of resources across the enterprise.

3. Specialties that utilize imaging but do not have a traditional imaging workflow can easily provision and enable one using the EI infrastructure. These specialties can create orders based or encounters-based workflows depending on requirements. These workflows will capture data that was previously only available locally and integrate it into the enterprise (EMR).

- Unnecessary duplicative imaging procedures
- Duplicative imaging infrastructure in departments using imaging (examples such as GI, dermatology, wound care, pulmonology)
- Optimal use of resources across the enterprise (e.g., global worklist for all radiologists in the enterprise)
- Centralized image storage infrastructure instead of a costly distributed infrastructure
- Centralized maintenance of unified imaging IT infrastructure

## Step 6: Show how EI Can Help Enhance Patient Experience

Imagine having the ability to offer your patients complete access to view their own image record. This would go a long way towards improving patient experience and can result in better patient retention.

From the perspective of staff productivity and operating costs, this can significantly reduce:

- The human and supply resources required to maintain workflows requiring burning imaging exams to CD/DVDs
- Mailing of CD/DVDs to external organizations and providers, including overnight, same-day and courier deliveries
- Turn-around times for final imaging results awaiting prior exams for comparison
- The human resources required to maintain workflows requiring uploading imaging exams from CD/DVDs
- The human resources required to walk the CD/DVDs provided by a clinic patient to the nearest radiology department, which can sometimes require walking to a different building or utilizing a courier service to deliver to a different campus

From the perspective of a patient, providing patient access to their own image record offers a progressive, yet increasingly expected approach in a rapidly evolving world of social media and online access driving consumer choices.

In June 2016, a Connected Care and the Patient Experience survey was conducted by Kelton Global; 94% of patients feel their medical information and records should be centrally stored and electronically accessible, with a notable dissatisfaction with current limitations rendering them unable to access and share their own health records [4].

Organizations should also consider other factors contributing to enhanced patient experience during their clinical and emergency visits, including:

- The ability for images to be received electronically and reviewed by a specialist or emergency care provider, prior to the patient arriving. Not only can this help improve outcomes for emergent care but also reduces the possibility of additional radiation exposure and insurance rejection for reimaging.
- Offering patients more time with their provider, due to a decrease in provider time and effort trying to locate previous or current imaging and results.

## Step 7: Enhance the Work Life of Caregivers

EI provides value to your providers, including the ability to access multidisciplinary imaging in a single “click” and workflow enhancements such as supporting encounter-based imaging without requiring orders [5].

Imaging acquired outside radiology and cardiology departments is often performed by clinical care providers whose primary role is not imaging, such as medical assistants, nurses, and even the physician. Greater emphasis on intuitive workflows is ultimately important within these specialties [6]. Attempting to implement traditional order-based imaging workflows or tying imaging modalities used in these areas to desktop dependence, frequently, results in provider frustration and non-compliance.

When capturing photos, scope video waveforms and other clinical multimedia within visit or procedure-based specialties such as dermatology, otolaryngology, or surgery, it is not intuitive for providers to pause patient assessment and care to place an order solely for proper compliance with a workflow.

Manual workflow steps resulting from poor interoperability, such as connecting a camera to PC to download images, introduce workflow gaps usually addressed once the provider has time to complete. This workflow can be wrought with errors and non-compliance, only sporadically completed.

Accessing images, previously acquired using non-intuitive and gap prone workflows, can further increase provider frustration and reduce efficiencies. Searching through a network directory of stored photos for a photo taken on a patient 6 months ago, to determine if a skin lesion has grown, requires time and additionally creates data security concerns. Equally inefficient and frustrating is searching through an image, or external media, archive with limited or no information labels to indicate what the clinical media is, or when it was performed.

Enterprise imaging, through the delivery of enhanced workflows, including encounter-based imaging, alleviates many of these challenges by:

- Offering intuitive workflows to visit and procedure-based imaging specialties, improving provider satisfaction and compliance
- Blending mobile and desktop access and acquisition
- Providing capture devices with modality worklists and patient schedules comprised of appropriate metadata for content indexing within the image archive and unified with the EHR record
- Validating images against the patient and EHR-associated visit, effectively linking the images to the appropriate patient encounter
- Eliminating the need for manual entry and workflows and contributing to errors, non-compliance and dirty data
- Scaling across multiple service lines for application with varied clinical images and multimedia such as photos, scope video, and point-of-care ultrasound
- Mitigating medico-legal issues [7] and patient care challenges presented if image records are inaccessible or unavailable
- Improving economies of scale and interoperability limitations associated with multiple image archives and the rapid increase in non-DICOM content [8]
- Complimenting the patient longitudinal record in the EHR, ultimately enhancing value for care plan decisions

## Step 8: Develop EI Governance

Governance is the keystone to any transformation process driving enterprise change. One transformation is the creation of an enterprise-wide, multi-department image management strategy that will align IT technology and clinical imaging initiatives across the healthcare environment. The SIIM-HIMSS EI workgroup published a white paper on EI governance in 2017 [9].

**Fig. 1:**
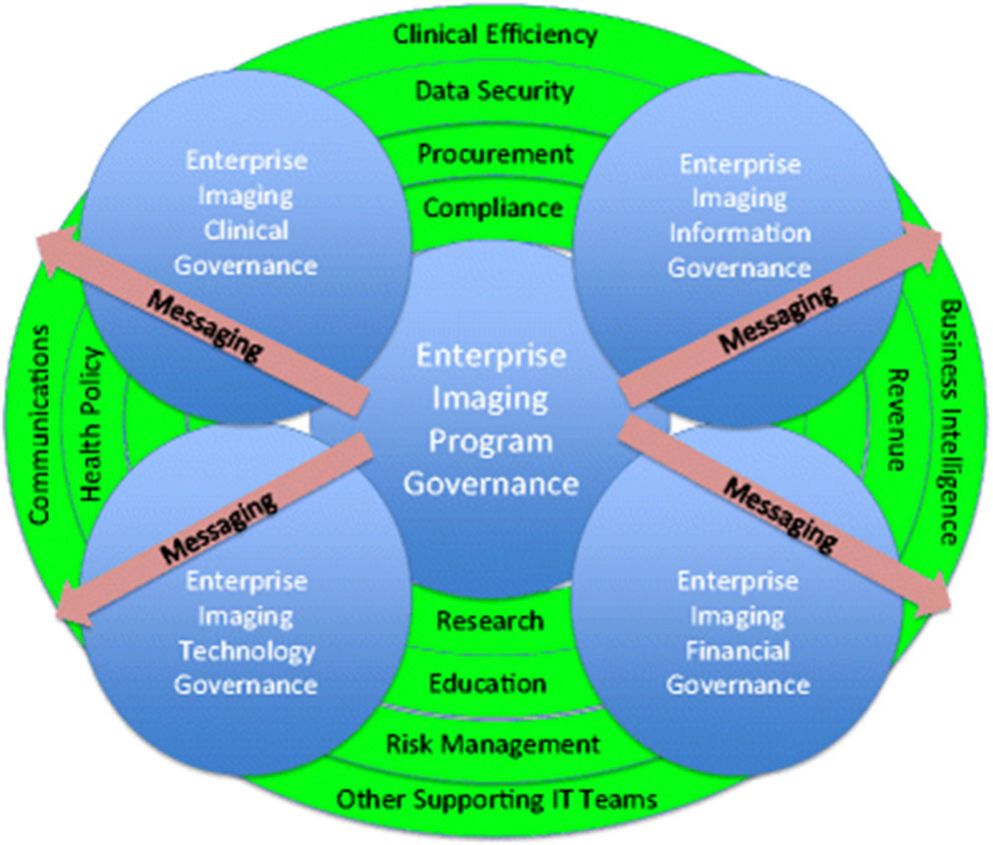
The integrated nature of EI governance: many non-imaging considerations impact EI decision-making in all five areas of focus

EI governance is an emerging need in health enterprises today. This white paper highlights the decision-making body, framework, and process for optimal EI governance inclusive of five areas of focus: program governance, technology governance, information governance, clinical governance, and financial governance. It outlines relevant parallels and differences when forming or optimizing imaging governance as compared with other established broad horizontal governance groups, such as for the electronic health record. It is intended for CMIOs and health informatics leaders looking to grow and govern a program to optimally capture, store, index, distribute, view, exchange, and analyze the images of their enterprise (Fig. 1).

The EI education workgroup recommends the EI advocates to study this whitepaper and familiarize themselves with the content which provides in-depth information to create an EI governance strategy. [9]

## Step 9: Implement an EI Project

The first decision to make as an enterprise is who do you want to be? If the decision is to be innovative and have a fully image-enabled EHR to facilitate better patient care, then the hard work begins. Typically, an informatics expert from an imaging-heavy department, such as radiology, initially sees the need and spearheads the initiative. The champion gives an EI presentation to the executive staff in an effort to educate, inform, and clarify any misconceptions. The most significant misconception is that EI mostly benefits radiology or it is a radiology project and not an enterprise initiative. The most prominent mistake I have seen multiple times is the failure to not consider EI in the early steps of replacing or upgrading the EHR. When an EHR replacement stretches the IT staff, it is challenging to add-on EI in the middle of the process or during the EHR optimization phase. Concurrent planning works best and similar resources such as a physician or nurse informatics groups can be leveraged for both initiatives. Once approval for the project is secured, form a small team of leaders to start the preplanning.

There are many paths to begin the journey of implementing an EI project. To obtain more information, attend a national conference such as the Radiological Society of North America (RSNA) or the Healthcare Information and Management Systems Society (HIMSS) or the Society for Imaging Informatics in Medicine (SIIM). The major vendors are present who can demonstrate their products and answer any questions. A few companies will provide a return on investment projections or recommend their consulting arm to perform a full review of the organization. Secondly, research and rankings of the top vendors are available from data analytic platforms such as KLAS. These reports can help guide you to which vendors to visit at RSNA, SIIM, or HIMSS. Lastly, independent consultants can be hired to perform a thorough analysis of your current state of imaging and guide you through the process to your desired state.

Meanwhile, having a better idea for your current imaging footprint will guide future enterprise decisions. Our IT staff compiled a list of over twenty different imaging applications installed at our institution. More importantly, knowing the maintenance costs, contract expirations, and end of life dates will assist with vendor selection. To lessen the workload and improve efficiencies, consolidating applications should be on the roadmap. Prioritization of heavy imaging departments might suggest that only vendors with robust pathology, radiology, and cardiology PACS and software solutions be considered. For example, sophisticated cardiology software can eliminate redundancies such as individual niche cardiac MR image management, an echocardiogram program, and an EKG application. Pathology departments may convert to whole slide digital imaging, and this needs to be supported by an EI VNA.

The first few steps include forming a steering committee, developing a meeting schedule, establishing goals (short, medium, and long-term), and agree on the logistics of a kick-off meeting. Weekly status reports are useful to keep the major stakeholders engaged and informed. Short-term goals may include conducting interviews to determine workflows and the current state of EI. Furthermore, short-term goals may provide the opportunity for quick wins such as improved efficiencies with changes to existing workflow.

EI is a strategic goal which contains strategies, initiatives, and workflows. EI is for entire healthcare, enterprise wide to consistently and optimally capture, index, manage, store and distribute, view, exchange, and analyze all clinical imaging and multimedia with the end goal being able to enhance the EHR. Moving from the current state to the future state can be challenging. However, with the right leaders, an excellent roadmap, and symbiotic vendor relations, the journey will be rewarding.

## Step 10: Understand Cybersecurity for EI

In recent years, we have seen a staggering increase in cybersecurity attacks directly impacting healthcare delivery organizations.

In March 2018, their Internet Security Threat Report [10] Symantec states: “From the sudden spread of WannaCry and Petya/NotPetya, to the swift growth in “coin miners,” 2017 provided us with another reminder that digital security threats can come from new and unexpected sources.” Further, the Ponemon Institute reported already in 2016 that healthcare providers are in the crosshairs of cyber attackers [11].

The trend of hospital consolidation has created the emergence of EI service lines which consist of multiple departments in different hospitals belonging to an Integrated Delivery Network or IDN. As an example, the dermatology and imaging service line can now consist of the different dermatology and radiology departments in the different hospitals that once did not belong to the same IDN organization. These service lines hold the promise to provide higher quality service at a lower cost to patients and their care teams due to efficiency gains and savings made through economies of scale. The downside is that such organizational structure can make cybersecurity threats even more acute and potentially more impactful.

Why? All computer-based equipment and various applications such as image viewers in the service lines are becoming more and more interconnected or consolidated into a centralized system. Through the IT network infrastructure connecting the different service lines locations in the healthcare enterprise, cyber-attacks now have another available vector to quickly spread malware and compromise these service lines operations.

### Cybersecurity for EI Initiatives

EI deployments are augmenting the risk of exposure to a computer virus or other malware infections and significant threats including denial of service, phishing e-mails, phishing websites, social phishing, brute forcing, and FTP server compromises. As already mentioned in this white paper, numerous departments are using imaging in the visible light spectrum as diagnostic, procedural, and evidence data. Images from endoscopes, point and shoot cameras, specialized medical cameras, and image flatbed scanners were acquired and stored completely offline, either as digital images, e.g., in jpeg format on a local PC or plain hard copy in a patient folder, and were not connected to the hospital’s intranet. EI changes this paradigm completely. Caregivers will send their digital images straight into the IDN’s intranet and these images or videos will be routed to their repository destination: the IDN’s data center where all patient’s images are stored in the enterprise vendor neutral central archive and digitally linked to the patient’s electronic health record. This increases the risk of a computer virus being introduced into the EHR.

Medical imaging acquisition devices, like all computer-based systems, are subject to cybersecurity risks that might harm patient safety and even patient lives if a hacker interferes with the safe operation of an imaging device, PACS, EHR, or any other IT-based system [12] by blocking access or falsifying the content. As a consequence, vital patient data may not be available for caregivers. Then, there is the risk that hackers can steal the patient’s medical records gaining access to electronic protected health information (ePHI). Last but not least, hackers may also steal the user’s or patient’s log-in credentials allowing for unauthorized access to ePHI.

The NEMA MITA Cybersecurity Task Force (CTF) published a white paper cybersecurity for medical imaging [12] that identifies a set of best practices and guidelines that both medical imaging manufacturers and the user community can implement to minimize the possibility that bugs, malware, viruses, and exploits can be used to negatively impact patient safety, product operation, or compromise ePHI and patient safety. The paper was developed in collaboration with the American College of Radiology, a professional organization for medical imaging.

The guidelines and best practices described in this document, aimed at radiology departments, can, to a great extent, also be applied to EI initiatives. The CTF team came to a major conclusion: advancing cybersecurity measures within healthcare and public health relies on a whole community approach, requiring manufacturers, installers, service staff, and caregivers to accept shared ownership and responsibility. The FDA agrees with NEMA MITA that this problem requires a collaborative approach. [13]

### Prevention of Cybersecurity Breaches

We all know the axiom “an ounce of prevention is worth a pound of cure.” Prevention of cybersecurity breaches will require cyber awareness. The CTF white paper takes this approach and refers to standards and best practices in cybersecurity. However, a truly robust cybersecurity plan can be only achieved when cybersecurity processes are clearly defined and effectively and consistently followed by well-trained departmental staff. It is all about the trinity of people, processes, and technology.

IBM stated already in their 2014 Cyber Security Intelligence Index [14] that over 95% of all incidents investigated recognize “human error” as a contributing factor.

The CTF white paper also discusses the HIMSS/NEMA HN 1-2013 Manufacturer Disclosure Statement for Medical Device Security (MDS2) [15]. MDS2 was developed by MITA and members of the HIMSS Medical Device Security Task Force in collaboration with multiple industry associations, government agencies, and other stakeholders. This standard specifies a device manufacturer’s model-specific description of a device’s ability to maintain/transmit electronic protected health information (ePHI) and the security features associated with the device. MDS2 provides most of the required information if you decide to perform an ISO 80001-1-based cybersecurity risk management audit in collaboration with your IT department.

We all need to be aware that cybersecurity prevention is based on an ecosystem of people, processes, and technologies. Imaging staff must be aware of cybersecurity threats and best-in-class practices. Imaging service lines need to build their cybersecurity strategy and operational plans in close collaboration with IT departments.

## Conclusion

It is probably clear to the readership that substantial resources will be needed to reap the benefits of EI for patients, caregivers, and the healthcare provider organization. The authors feel that this white paper addresses the many points that need to be taken in consideration when developing a presentation to the leadership of a healthcare organization and how to answer many detailed questions that C-suite advisory bodies may ask. Further, the information can also be used to fuel EI discussion groups for different audiences in the Integrated Delivery Network who will become important stakeholders in the EI strategy development. The authors have all substantial experiences in EI, from a perspective of management strategy, governance, clinical, financial, and technical experience, and are all involved in “real-world” EI implementations. Feel free to contact the HIMSS-SIIM EI workgroup.
